# Influence of the Emulsifier Sodium Caseinate–Xanthan Gum Complex on Emulsions: Stability and Digestive Properties

**DOI:** 10.3390/molecules28145460

**Published:** 2023-07-17

**Authors:** Huan Huang, Yan Tian, Xinpeng Bai, Yumiao Cao, Zihuan Fu

**Affiliations:** 1 School of Food Science and Engineering, Hainan University, No. 58 Renmin Avenue, Haikou 570228, China; 18279005573@163.com (H.H.); tianyan@hainanu.edu.cn (Y.T.); mmcao94wsx@163.com (Y.C.); fzh17793171572@163.com (Z.F.); 2 Engineering Research Center of Utilization of Tropical Polysaccharide Resources, Ministry of Education, Hainan University, No. 58 Renmin Avenue, Haikou 570228, China; 3 Haikou Zhisu Biological Resources Research Institute Co., Ltd., Haikou 570203, China

**Keywords:** co-adsorption, layer adsorption, nanoemulsion, stability, digestion

## Abstract

In this study, virgin coconut oil (VCO) nanoemulsions were prepared by ultrasonication using a sodium caseinate (SC) and xanthan gum (XG) complex as an emulsifier. The stability and digestion characteristics of SC/XG–VCO emulsions formed by co-adsorption and SC–VCO–XG emulsions formed by layer adsorption were compared. The stability of the two emulsions was studied under different pH, ionic strength, heat treatment, freeze–thaw cycles, and storage conditions, and the droplet size and zeta potential were used as indicators to assess the stability. In addition, the stability of oxidation and the digestive properties of both emulsions were studied. It was found that the SC–VCO–XG emulsions had better environmental stability, oxidative stability, storage stability, and digestibility compared to SC/XG–VCO emulsions. This study has shown that the formation method of protein–polysaccharide stabilized emulsions has an impact on the stability and digestibility properties of the emulsions, and that the emulsion carriers constructed by layer adsorption are more suitable for subsequent industrial production and development.

## 1. Introduction

A nanoemulsion consists of an oil phase, an aqueous phase and a surfactant (emulsifier) with droplet sizes of between 10 and 1000 nm [1]. Recently, nanoemulsions have been widely used in food, cosmetic and pharmaceutical applications [2]. However, nanoemulsions are thermodynamically unstable, and stabilizers such as emulsifiers and thickeners promote their kinetic stability [3]. At present, most of the emulsifiers used are active surface molecules that are attracted to the surfaces of oil droplets during homogenization and inhibit their aggregation by reducing interfacial tension and the resulting formation of a protective film on their surfaces [4]. Many techniques have been used to homogenize emulsions, such as ultrasound, high-pressure homogenization, microjet, etc. [5]. The main principle of the emulsification process is the continuous fragmentation of the dispersed phase droplets until a steady state is reached [6,7].

Natural emulsifiers that are commonly used are proteins and polysaccharides. Proteins could be adsorbed to the oil–water interface and prevent aggregation and agglomeration of droplets through electrostatic repulsive forces and the spatial site resistance effect, thus stabilizing the emulsion [4]. However, protein stabilized emulsions are relatively less stable under various external environments (pH, ionic strength, heat treatment, freeze–thawing, etc.). To improve stability, it is possible to add polysaccharides to increase the viscosity of the continuous phase and to increase the thickness of the interfacial layer by adsorption on the surface of the protein-coated droplets [8,9]. For example, Xu et al. [10] found that the pH, salt and thermal stability of rice gluten (HRG) emulsions could be improved by the addition of xanthan gum. Cabezas et al. [11] found that the freeze–thaw stability of whey protein isolate–soluble soy polysaccharide mixtures stabilized emulsions was better than that of whey isolate protein alone. Sodium caseinate (SC) is the sodium salt of casein, which has better emulsifying activity than vegetable proteins such as soy and pea proteins. For example, Ho et al. [12] found that lycopene emulsions stabilized by sodium caseinate had better physicochemical stability than soybean isolate protein or pea isolate protein. Xanthan gum (XG) is a polysaccharide, commonly used as a stabilizer, with high viscosity and strong shear-thinning properties, which can be used in combination with protein to improve the stability of emulsions. For instance, Sun et al.’s study [13] found that the oxidative stability of protein emulsion systems was improved with the addition of xanthan.

Protein–polysaccharide complexes stabilized emulsions are formed by co-adsorption or layer adsorption. During co-adsorption, protein and polysaccharide are simultaneously adsorbed onto the oil–water interface to stabilize the emulsion. During layer adsorption, the protein is adsorbed onto the oil–water interface, and then the polysaccharide adsorbed onto the protein layer is added to form a bilayer, and the increase in the thickness of the interfacial layer stabilizes the emulsion by increasing the electrostatic repulsive force, to inhibit droplet aggregation [14]. Currently, many studies have focused on the effects on emulsion stability of protein–polysaccharide interactions and their adsorption onto the interfaces. It has been found that the β-lactoglobulin–pectin complexes stabilized emulsions prepared by layer adsorption exhibited good stability against ionic strength and pH treatment [15,16]. Ray et al. [17] found that soy whey protein-soluble soybean polysaccharides co-adsorbed on the surface of oil droplets to form an interfacial adsorption layer to improve emulsion stability. However, less information is available on comparative studies of the stability and digestive properties of emulsions prepared by co-adsorption or layer adsorption.

In this study, the emulsions were prepared by two methods (co-adsorption and layer adsorption) using sodium caseinate (SC)–xanthan gum (XG) complexes as emulsifiers. The emulsions’ stability against external environments (pH, ionic strength, heat treatment, freeze–thawing, and storage, etc.) and digestibility properties of emulsions were characterized. This study investigated the influence of the adsorption strategy of emulsifiers on emulsification stability and emulsion digestion characteristics and provided a theoretical basis for preparing a stable emulsification system.

## 2. Results and Discussion

### 2.1. pH Stability

**Figure 1:**
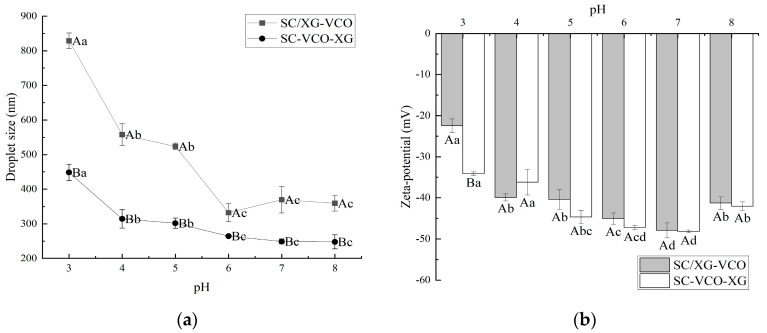
Effect of pH on the droplet size (**a**) and zeta potential (**b**) of coconut oil nanoemulsions (different lowercase letters represent significance within the group; different capital letters represent significance between the groups).

The droplet sizes of both emulsions decreased significantly with increasing pH. Under low pH conditions, the electrostatic repulsion between emulsion droplets was less, resulting in droplet aggregation, and thus presenting a larger droplet size. Yan et al. [18] also found that soy protein–chasaponin stabilized emulsions increased in droplet size under low pH conditions, due to weakened electrostatic repulsion. Meanwhile, it can be seen from Figure 1a that the emulsions formed by SC–XG layer adsorption had smaller droplet sizes, indicating that layer adsorption was more favorable to improve the emulsifying activity of protein–polysaccharide, and therefore formed a smaller droplet size emulsion. This was due to the added polysaccharide providing sufficient spatial site resistance and electrostatic repulsion to prevent the emulsion from aggregating into large particles. It has been shown that smaller droplet sizes facilitate to the maintenance of stability in emulsions. Therefore, it was hypothesized that the emulsions formed by protein–polysaccharide layer adsorption could exhibit better stability, especially in neutral environments. Wei et al. [19] found that the complex formed by sodium caseinate and chitosan increased the stability of the emulsion for heat treatment situations by increasing the thickness of the adsorption film through layer-wise adsorption on the surface of oil droplets.

As shown in Figure 1, the net charge of the emulsion showed a gradual increase with increasing pH. It was shown that the higher net charge of the emulsion provided electrostatic repulsion, that facilitates its stability. Therefore, it was presumed that the emulsion was more stable in a neutral environment. Zhao et al. [20] found that lactoferrin–beet pectin stabilized emulsions were stable under neutral conditions because of increased electrostatic repulsion between droplets due to the adsorption of anionic polysaccharides.

### 2.2. Ionic Strength Stability

**Figure 2:**
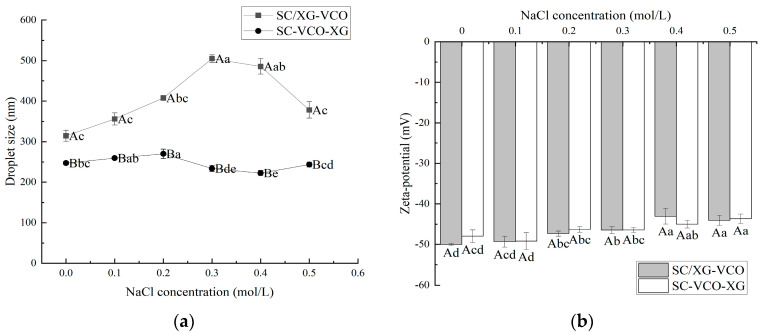
Effect of NaCl concentration on the droplet size (**a**) and zeta potential (**b**) of coconut oil nanoemulsions (different lowercase letters represent significance within the group; different capital letters represent significance between the groups).

From Figure 2a, it can be seen that the droplet sizes of both emulsions showed an increasing trend with the increase in ion concentration. This may be because the addition of salt weakened the electrostatic attraction between protein and polysaccharide, and some of the polysaccharides were desorbed from the surface of the droplet, leading to the aggregation of the emulsions, hence the trend of increasing droplet sizes. Zhao et al. [20] found that the droplet sizes of lactoferrin–soybean soluble polysaccharide stabilized emulsions increased with increasing ionic strength due to the desorption of polysaccharides from the emulsion droplet’s surface, leading to the emulsions’ aggregation. Also, the droplet sizes of SC–VCO–XG emulsions were significantly smaller than those of SC/XG–VCO emulsions over the entire NaCl concentration range. The added polysaccharide provided sufficient spatial site resistance and reduced the van der Waals attraction between emulsion droplets, slowing down the rate of emulsion aggregation. Zhao et al. [20] found that the complex formed by lactoferrin and beet pectin increased the thickness of the interfacial layer by layer-wise adsorption on the surface of oil droplets, improving the stability of emulsion to ionic strength.

The absolute zeta potential values of both emulsions showed a decreasing trend with increasing salt ion concentrations (Figure 2b). This was due to the electrostatic screening effect triggered by adding NaCl. Azarikia et al. [21] found that the absolute value of zeta potential of whey protein–astragalus gum stabilized emulsions increased with increasing NaCl concentration due to the electrostatic screening effect.

### 2.3. Thermal Stability

**Figure 3:**
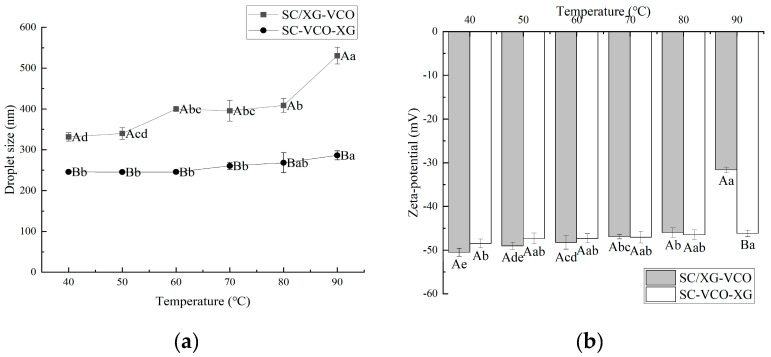
Effect of heat treatment temperature on the droplet size (**a**) and zeta potential (**b**) of coconut oil nanoemulsions (different lowercase letters represent significance within the group; different capital letters represent significance between the groups).

From Figure 3a, it can be seen that the droplet sizes of the emulsion showed a gradual increase with increasing heating temperature. A similar phenomenon was found by Chen et al. [22] when they investigated the effect of heating treatment on the droplet sizes of whey protein–xanthan gum stabilized emulsions. Also, emulsions formed by layer adsorption had smaller droplet sizes under heat treatment conditions. Polysaccharide incorporation into protein stabilized emulsions protected the protein from heat and provided sufficient electrostatic repulsion and spatial site resistance to avoid aggregation of the emulsions. When Azarikia et al. [21] investigated the thermal stability of whey protein–xanthan gum stabilized emulsions, it was found that the emulsions formed by layer adsorption had improved stability under a heated environment due to the polysaccharides adsorbed in the outer layer protecting the protein from heat.

The absolute value of the zeta potential of the emulsions tended to decrease gradually with increasing heating temperature, and the degree of potential change was smaller for SC–VCO–XG emulsions (Figure 3b). The decrease in the absolute value of the zeta potential weakened the electrostatic repulsion between the emulsion droplets, which was detrimental to the stability of the emulsions. Therefore, it was hypothesized that emulsions formed by protein–polysaccharide layer adsorption presented better stability under heat treatment conditions. Zang et al. [23] found that pectin adsorbed on the surface of rice bran protein-coated droplets increased the electrostatic repulsion between droplets, leading to improved stability of emulsions under heat treatment conditions.

### 2.4. Freeze-Thaw Stability

**Figure 4:**
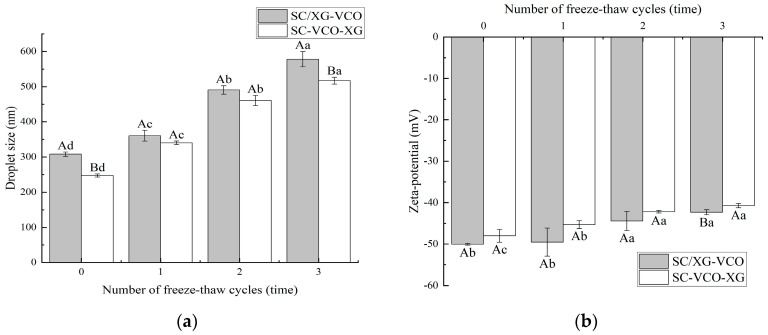
Effect of freeze–thaw cycles on the droplet size (**a**) and zeta potential (**b**) of coconut oil nanoemulsions (different lowercase letters represent significance within the group; different capital letters represent significance between the groups).

The droplet sizes of the emulsions increased with the number of freeze–thaw cycles (Figure 4a). A similar trend was found by Donsì et al. [24] when they studied the freeze–thaw stability of emulsions stabilized by lecithin and modified starch. Also, the droplet sizes of SC–VCO–XG emulsions were smaller than those of SC/XG–VCO emulsions. The smaller the droplet sizes, the better the stability of the emulsions, so we speculated that the freeze–thaw stability of SC–VCO–XG emulsions was better. This was due to the increased thickness of the interfacial layer of the emulsion formed by layer adsorption, which avoids agglomeration of the droplets. Donsì et al. [24] found that lactoferrin and beet pectin increased the thickness of the interfacial layer by layer adsorption on the surface of the oil droplets, which improved the stability of the emulsion during freeze–thaw.

As shown in Figure 4b, the absolute value of the zeta potential of both emulsions decreased with the increase in the number of freeze–thaw cycles, which may be due to the partial oil–water separation of the emulsions during the freeze–thaw process and the decrease in the concentration of the emulsion layer, resulting in a reduction of the number of charges they carry [25]. A similar phenomenon was found by Hou [25].

### 2.5. Oxidative Stability

**Figure 5:**
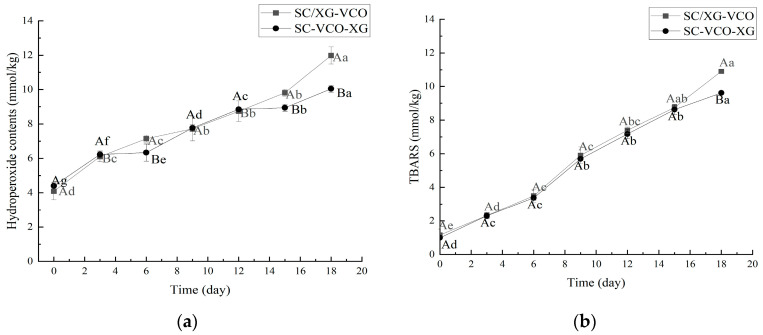
Hydroperoxides (**a**) and TBARS (**b**) of coconut oil nanoemulsion stored at 60 °C in the dark for up to 18 days (different lowercase letters represent significance within the group; different capital letters represent significance between the groups).

As shown in Figure 5a, the hydroperoxide content of both emulsions increased with storage time. After 18 days of storage, the hydroperoxide content was SC/XG–VCO emulsion > SC–VCO–XG emulsion, which was similar to the results of Liao et al. [26]; these authors found that the hydrogen peroxide value of the perilla oil emulsion formed by layer adsorption was lower than that of the emulsion formed by co-adsorption because the increased thickness of the interfacial layer of the emulsion reduced the contact between the oil and the pro-oxidants in the air, resulting in a slower rate of oxidation of the oil.

As shown in Figure 5b, the trend of the TBARS value was consistent with the direction of the hydroperoxide value. The antioxidant effect of the SC–VCO–XG emulsion was better than that of the SC/XG–VCO emulsion, which may be due to the increased thickness of the interfacial layer of the SC–VCO–XG emulsion, which reduced the contact between coconut oil and the pro-oxidant and slowed down the oxidation of coconut oil [26].

### 2.6. Storage Stability

**Figure 6:**
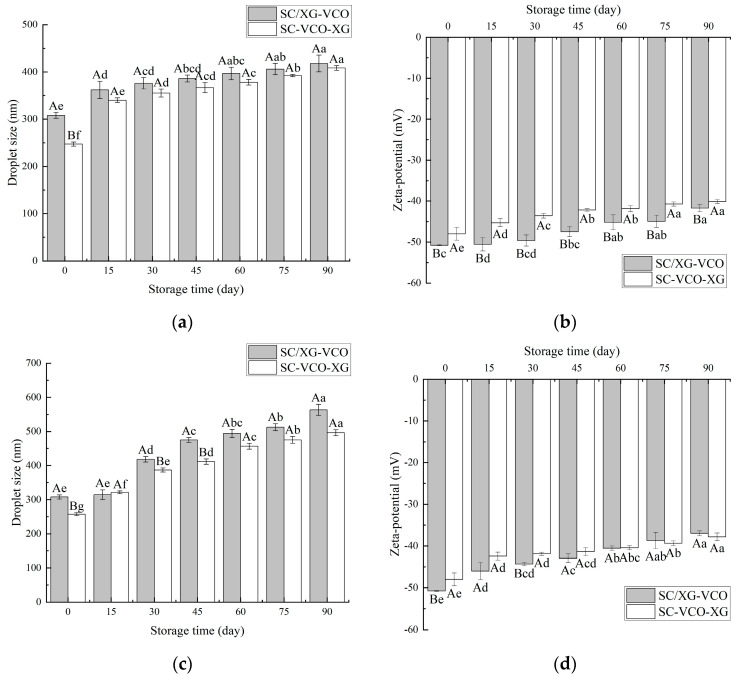
Changes in droplet size and zeta potential of emulsions during storage at 4 °C (**a**,**b**) and room temperature (**c**,**d**) (different lowercase letters represent significance within the group; different capital letters represent significance between the groups).

As shown in Figure 6a, the droplet sizes of both emulsions increased with time, and the droplet sizes measured at 15 days of storage were significantly larger for both emulsions. This occurred because, when the emulsions were at low temperatures, the encapsulated coconut oil crystallized and large droplets appeared, increasing the droplet sizes of the emulsions [24]. A similar trend was found when Sun [27] studied the droplet size variation of soybean isolate and tea saponin stabilized emulsions at 4 °C. From Figure 6b, it can be seen that the net charge of the emulsions decreased with increasing storage time, but the absolute value of SC–VCO–XG emulsions decreased less. The decrease in the absolute value of the zeta potential made the emulsions less stable [28], which indicated that the storage stability of SC–VCO–XG emulsions at 4 °C was better.

As shown in Figure 6c, the droplet sizes of the emulsions increased significantly with the storage time. This may be attributed to the flocculation and coalescence of emulsion droplets that occurred during storage [29]. The droplet size of the SC/XG–VCO emulsion increased by 255.64 nm, and the droplet size of the SC–VCO–XG emulsion increased by 249.04 nm, indicating that the storage stability of the SC–VCO–XG emulsion was better than that of the SC/XG–VCO emulsion. This was due to the xanthan gum in the SC–VCO–XG emulsion, that can provide sufficient electrostatic repulsion and spatial site resistance and also increase the viscosity of the emulsion; this could reduce the probability of aggregation of the emulsion droplets due to collision during the movement of the emulsion droplets in the storage period, thus reducing the rate of droplet size increase [29]. Zhang [29] also found that emulsions formed from egg white protein–chitosan complexes by layer-by-layer adsorption had better storage stability. As shown in Figure 6d, the absolute value of the zeta potential of the emulsions showed a decreasing trend, which was consistent with the trends of the droplet sizes, and the total value of the zeta potential of the SC–VCO–XG emulsion decreased less. This further indicated that the room temperature storage stability of the SC–VCO–XG emulsion was better. Overall, the storage stability of the emulsions formed by layer adsorption was better.

### 2.7. Digestive Properties

#### 2.7.1. Changes in Droplet Size and Zeta Potential during In Vitro Digestion

**Figure 7:**
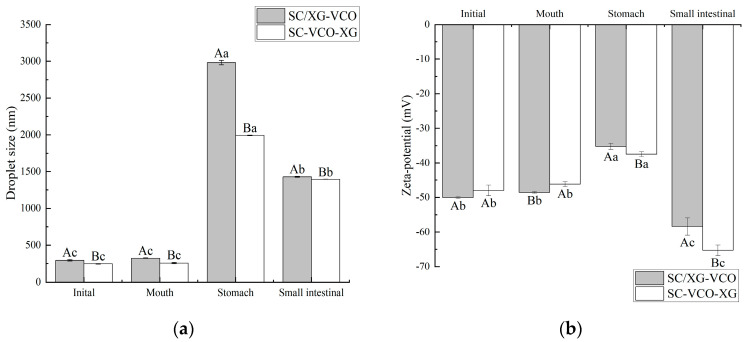
Changes in droplet size (**a**) and zeta potential (**b**) of coconut oil nanoemulsion during simulated digestion (different lowercase letters represent significance within the group; different capital letters represent significance between the groups).

During in vitro digestion, the droplet size and zeta potential of the emulsions were determined after each digestion stage (Figure 7). Initially, the droplet size of the SC–VCO–XG emulsion was smaller compared to the SC/XG–VCO emulsion (*p* < 0.05), which may be attributed to the aggregation of sodium caseinate and xanthan gum and formation of larger droplets under electrostatic or hydrophobic action before homogenization. The droplet size and potential of the two emulsions did not change significantly after oral digestion (*p* > 0.05); this may be because of the absence of surface-active ingredients to replace the emulsifier in the simulated saliva, the short duration of the simulated oral digestion and the lack of enzymes to break down the emulsions. The structure of the emulsions was not damaged and, in turn, the aggregation of emulsion droplets did not occur.

After stomach digestion, the droplet size and potential fluctuations of the SC–VCO–XG emulsion were smaller than those of the SC/XG–VCO emulsion (Figure 7), indicating that emulsions formed by layer adsorption were more stable under gastric digestion conditions. The increase in droplet size was attributed to the presence of pepsin in the simulated gastric juice, which hydrolyzed the proteins at the emulsion interface and disrupted the droplet structure, increasing the droplet size of the emulsion [30]. The reduction in potential can be attributed to the low pH of the simulated gastric juice, as the electrostatic repulsion between the emulsion’s droplets weakens under low pH conditions, reducing the absolute value of the zeta potential [31].

After digestion in the small intestine, the droplet sizes of emulsions formed by layer adsorption were significantly smaller than those emulsions formed by co-adsorption (*p* < 0.05) (Figure 7a), indicating that emulsions formed by layer adsorption were more stable under enteric digestion conditions. The smaller droplet size was due to a change in the ionic composition of the digestive fluid when moving from the gastric to the enteric digestion phase, where the bile salts had an emulsifying effect on the oils and fats, resulting in smaller droplet sizes of the emulsion [32]. During the intestinal digestion phase, the absolute value of the emulsion potential decreased significantly (Figure 7b), which was due to the pH of the system being around 7.0 during the simulated intestinal digestion phase. This facilitated the adsorption of negatively charged lipids and bile salts on the surface of the oil droplets or micelles, leading to an increase in anions in the system after simulated intestinal digestion.

#### 2.7.2. Digestibility

**Figure 8:**
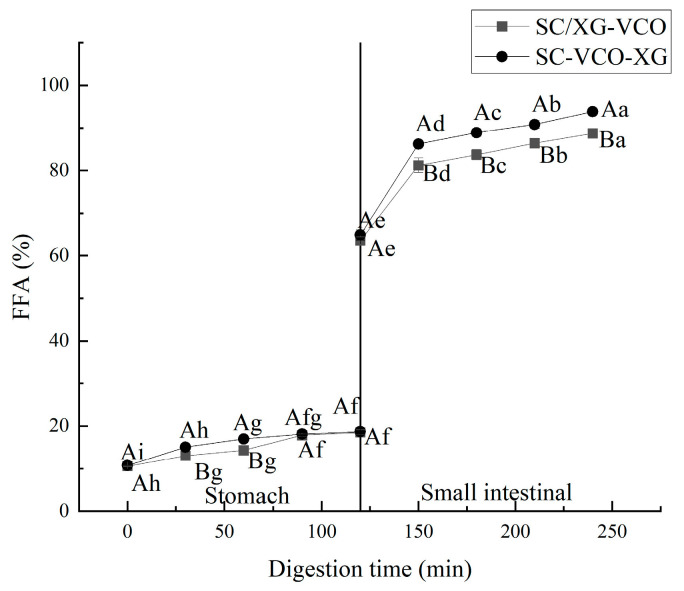
Free fatty acid release rate of emulsions (different lowercase letters represent significance within the group; different capital letters represent significance between the groups).

The free fatty acid release rates of two emulsions were compared during gastrointestinal digestion, and the results are shown in Figure 8. The free fatty acid release rates of the two emulsions during simulated gastric digestion were relatively low for the transition to the simulated intestinal digestion phase, after which the release rates of free fatty acids increased rapidly (Figure 8). Liu et al. [33] found a similar trend when they studied the release rates of free fatty acids from oils and fats during gastrointestinal digestion. Moreover, it can be seen from the graph that the release rate of free fatty acids in the simulated gastric digestion phase was much lower than that in the intestinal digestion phase, indicating that digestion was mainly carried out in the small intestine. Also, it can be seen that the free fatty acid release rate of the SC–VCO–XG emulsion was higher than that of the SC/XG–VCO emulsion, which may be because the droplet size of the SC–VCO–XG emulsion was smaller than that of the SC/XG–VCO emulsion; digestibility of lipids, in turn, increases due to the decrease in the droplet size [34].

## 3. Materials and Methods

### 3.1. Materials

Virgin coconut oil (VCO) was generously provided by Haikou Zhisu Biological Resources Research Institute Co., Ltd. (Haikou, China). Sodium caseinate (SC, food grade) was purchased from Henan Wanbang Industrial Co., Ltd. (Shangqiu, China). Xanthan gum (XG, food grade) was obtained from Henan Wanbang Industrial Co., Ltd. (Shangqiu, China). All other reagents were of analytical grade, and deionized water was used in all assays.

### 3.2. Preparation of the Coconut Oil Nanoemulsion

Stock SC solution: The SC solution was obtained by dissolving 2 g of sodium caseinate (SC) in 100 mL of phosphate-buffered solution (PBS, pH 7.2–7.4) with continuous stirring for 2 h, then hydrated overnight in the refrigerator at 4 °C, followed by removal of insoluble material by centrifugation (6000 r/min, 15 min). Thimerosal (≤0.2 mg/mL) was added as a bacterial inhibitor. Stock XG solution: Xanthan gum (1 g) was dissolved in 100 mL of pH 7.2~7.4 PBS buffer, stirred at medium-high speed for 1.5 h, and then placed in a refrigerator at 4 °C.

In this study, two methods were used to prepare emulsions co-stabilized by SC and XG. The detailed preparation method was as follows:

SC/XG–VCO nanoemulsion: Stock SC and XG solutions were mixed (1:1 *v*/*v*), then the pH was adjusted to 7 with 0.1 mol/L HCl. Then, oil (15.0% *w*/*w*) was added to prepare coarse emulsions, using a high-speed shear emulsion homogenizer (Shanghai Huxi Industrial Co., Ltd., Shanghai, China) at 20,000 r/min for 3 min. Finally, nanoemulsions were obtained by treatment of coarse emulsion with an ultrasonic bath processor (Kunshan Ultrasonic Instruments Co., Ltd., Kunshan, China) at an output power of 480 W for 18 min. All nanoemulsions were cooled using an ice bath during homogenization to prevent excessive heating caused by ultrasonic treatment.

SC–VCO–XG nanoemulsion: SC solution was first mixed with coconut oil, homogenized at 20,000/min for 3 min using a high-speed shear emulsification homogenizer, sonicated at 480 W for 18 min, and then xanthan gum was added, using a high-speed shear emulsification homogenizer, for 1 min to obtain the SC–VCO–XG nanoemulsion.

### 3.3. Stability Experiments

#### 3.3.1. pH Stability

The newly prepared emulsions were placed in sample bottles, and the emulsions’ pH was titrated to 3~9 by adding 0.1 mol/L HCl or 0.1 mol/L NaOH; the droplet size and zeta potential were determined after standing for 24 h [35].

#### 3.3.2. Ionic Strength Stability

Using the method described by Jiang et al. [36], with suitable modifications, 0.1~0.5 mol/L NaCl solutions were configured, the emulsions were diluted to twice the original volume with different concentrations of NaCl solutions, and the droplet size and zeta potential were determined after standing for 24 h.

#### 3.3.3. Thermal Stability

The two newly prepared emulsions were put into sample bottles, heated in a water bath at 40~90 °C for 30 min, then removed and cooled to room temperature. The droplet size and zeta potential were measured after 24 h at room temperature [37].

#### 3.3.4. Freeze–Thaw Stability

The fresh emulsion was placed in a sample bottle, stored in a refrigerator at −20 °C for 24 h, then removed and kept at room temperature for 30 min. The freeze–thaw cycle was repeated three times and, after each cycle, the droplet size and zeta potential were measured [38].

#### 3.3.5. Oxidation Stability

The emulsions were kept at 60 °C for 18 d, and the amount of primary oxidation products (hydroperoxides) and secondary oxidation products (thiobarbituric acid reactive substances, TBARS) produced by the samples was measured every 3 d to determine the oxidative stability of the emulsions. The hydroperoxide content was determined by reference to Liao et al. [26]; the procedure was as follows: 0.3 mL of the emulsion was mixed with 15 mL of isooctane–isopropanol (3:1, *v*/*v*) mixture, vortexed for 20 s, and shaken 3 times with 20 s intervals between each shaking, followed by centrifugation at 5500 r/min for 10 min to obtain the oil phase. The upper organic phase was transferred into a 10 mL centrifuge tube, and 2.8 mL of methanol–butanol (2:1, *v*/*v*) mixture was added, followed by 15 µL of 0.144 mol/L ferrous solution and 15 μL of 3.14 mol/L ammonium thiocyanate solution, shaken well, placed in the dark for 20 min and the absorbance value was measured at 510 nm. The standard curve was plotted using a standard solution prepared from hydrogen peroxide. The standard curve equation was y = 0.0721x − 0.0156, R^2^ = 0.991, where y was the absorbance value, and x was a hydrogen peroxide concentration.

TBARS values were determined by referring to Zhang et al. [39], as follows: 1.0 mL of the emulsion was mixed with 2.0 mL of thiobarbituric acid test solution (consisting of 15.0 g trichloroacetic acid, 0.375 g thiobarbituric acid, 1.76 mL of 12 mol/L hydrochloric acid and 82.9 mL of ultrapure water), vortexed and mixed well. The absorbance value was measured at 532 nm, and the standard curve was plotted using a standard solution prepared from 1,1,3,3-tetra ethoxy propane. The standard curve equation was y = 0.2452x + 0.056 and R^2^ = 0.9958, where y was the absorbance and x was the TBARS value.

#### 3.3.6. Storage Stability

Fresh emulsion (10 g) was stored in sample bottles at 4 ℃ and room temperature, and the droplet size and zeta potential were measured every 15 days [40,41].

#### 3.3.7. Determination of Droplet Size and Zeta Potential

The droplet size was obtained using a Mastersizer 3000 (Malvern Instruments, Ltd., Malvern, UK). Wet dispersion was used, with PBS buffer (pH 7.2~7.4) as the dispersant. The specimens, including emulsion and dispersant, were put into the sample cell, and the shading rate of the specimens was measured at 10% or more (including 10%) by the principle of laser light scattering. The results were processed to obtain the particle size using Mastersizer 3000 software. The zeta potential of the nanoemulsions was determined using a Zetasizer Nano ZS90 analyzer (Zetasizer Nano ZS90, Malvern Instruments, Ltd., Great Malvern, UK). The refractive index of coconut oil was set to 1.46 and that of phosphate buffer to 1.33. The zeta potential was determined by diluting the emulsions 50 times with PBS buffer (pH 7.2~7.4) before the analysis, to avoid multiple scattering effects [41].

### 3.4. In Vitro Digestion Analysis

#### 3.4.1. Construction of In Vitro Digestion Model

The external digestion model was constructed with reference to Brodkorb et al. [42], consisting of three parts: mouth, stomach, and intestine. In vitro digestion experiments were conducted under light-proof conditions.

Simulated mouth digestion stage: A volume of 5 mL of the emulsion was taken to be digested, and 4 mL of SSF stock solution and 25 µL of 0.3 mol/LCaCl_2_ were added to it in turn. Then the pH was adjusted to 7 with 1 mol/L HCl or 1 mol/L NaOH, and distilled water was added to make the total volume of 10 mL. Finally, mucin was added to make the final concentration of mucin 3 mg/mL, and the mixture was stirred well and then shaken in a water bath at 37 °C for 10 min.

Simulated gastric digestion stage: SGF stock solution (8 mL), 5 µL of 0.3 mol/L CaCl_2_, and 1 mol/L HCl were added to the sample solution after oral digestion to adjust the pH to 3, and then distilled water was added to make the volume 20 mL, followed by lipase to a concentration of 4 mg/mL and pepsin to a concentration of 3.2 mg/mL, This mixture was stirred well and shaken in a water bath at 37 °C for 120 min.

Simulated intestinal digestion stage: SIF stock solution (16 mL), 40 µL of 0.3 mol/L CaCl_2_, and 1 mol/L NaOH were added to the sample solution after gastric digestion to adjust the pH to 7, and then distilled water was added to make the total volume 40 mL. Porcine bile salt and pancreatic enzyme were added to make the final concentrations of 5 mg/mL and 3.2 mg/mL, respectively, and then the mixture was stirred well and placed in a water bath at 37 °C for 120 min.

#### 3.4.2. Changes in Droplet Size and Potential during In Vitro Digestion

The different digestion stages were measured with their corresponding pH buffers, as described in Section 3.3.7.

#### 3.4.3. Determination of Free Fatty Acids

The degree of digestion of fats and oils was studied at different periods throughout the simulated digestion process. The release rate of free fatty acids (FFA) was measured at 0, 30, 60, 90, and 120 min of shaking after adding pepsin and pancreatic enzymes in the gastrointestinal phase. The method of FFA release rate was determined by using the method of Liu et al. [33]. Digested sample (1 mL) was added to 25 mL of ether/ethanol solution (ether: ethanol, 1:1, vol/vol), and several drops of phenolphthalein were added and titrated with 0.01 mol/L KOH solution to the endpoint.

(1) $$\mathrm{FFAs}\left(\%\right)=\frac{0.01V_{1}M_{\mathrm{lipid}}}{m_{\mathrm{lipid}}}\times 100\%$$

where FFAs: free fatty acid release rate, %; m_lipid_: the total mass of fat in the emulsion, g; V_1_: volume of KOH solution consumed, L; M_lipid_: average molecular weight of fat molecules, g/mol, which was obtained by measuring the average molecular weight of coconut oil of 648 g/mol by saponification of coconut oil.

### 3.5. Data Processing and Analysis

All experiments were repeated three times, and the results are shown as mean ± standard deviation. Analysis of variance was performed using IBM SPSS Statistics 23, to determine the significance of differences. Tukey’s test was used to determine significant differences between samples at the 5% level of significance (*p* < 0.05), and graphs were prepared using Origin 2021 software.

## 4. Conclusions

In this study, sodium caseinate and xanthan gum were compounded as emulsifiers and coconut oil emulsions were prepared by ultrasonication. The stability and digestibility of SC–VCO–XG emulsions were higher than those of SC/XG–VCO. This study has shown that the formation method of protein–polysaccharide stabilized emulsions has an impact on the stability and digestibility properties of the emulsions, and that the emulsion carriers constructed by layer adsorption are more suitable for subsequent industrial production and development. There are still some areas that need to be further evaluated in this study, such as interface rheology experiments.

## Data Availability

Data are contained within the article.

## References

[1] Jaiswal M., Dudhe R., Sharma P.K. (2015). Nanoemulsion: An advanced mode of drug delivery system. 3 Biotech.

[2] Solans C., Solé I. (2012). Nano-emulsions: Formation by low-energy methods. Curr. Opin. Colloid Interface Sci..

[3] Wei Y., Tong Z., Dai L., Ma P., Zhang M., Liu J., Mao L., Yuan F., Gao Y. (2020). Novel colloidal particles and natural small molecular surfactants co-stabilized Pickering emulsions with hierarchical interfacial structure: Enhanced stability and controllable lipolysis. J. Colloid Interface Sci..

[4] Albano K.M., Cavallieri Â.L.F., Nicoletti V.R. (2019). Electrostatic interaction between proteins and polysaccharides: Physicochemical aspects and applications in emulsion stabilization. Food Rev. Int..

[5] Kapiamba K.F. (2022). Mini-review of the microscale phenomena during emulsification of highly concentrated emulsions. Colloid Interface Sci. Commun..

[6] Masalova I., Kapiamba F., Tshilumbu N., Malkin A.Y. (2018). Shear Stability of Highly Concentrated Emulsions. Colloid J..

[7] Masalova I., Fabrice K.K., Tshilumbu N.N., George N., Malkin A.Y. (2018). Emulsification of highly concentrated emulsions—A criterion of shear stability. J. Rheol..

[8] Liu J., Verespej E., Alexander M., Corredig M. (2007). Comparison on the Effect of High-Methoxyl Pectin or Soybean-Soluble Polysaccharide on the Stability of Sodium Caseinate-Stabilized Oil/Water Emulsions. J. Agric. Food Chem..

[9] Wang S., Yang J., Shao G., Liu J., Wang J., Yang L., Li J., Liu H., Zhu D., Li Y. (2020). pH-induced conformational changes and interfacial dilatational rheology of soy protein isolated/soy hull polysaccharide complex and its effects on emulsion stabilization. Food Hydrocoll..

[10] Xu X., Luo L., Liu C., McClements D.J. (2017). Utilization of anionic polysaccharides to improve the stability of rice glutelin emulsions: Impact of polysaccharide type, pH, salt, and temperature. Food Hydrocoll..

[11] Cabezas D.M., Pascual G.N., Wagner J.R., Palazolo G.G. (2019). Nanoparticles assembled from mixtures of whey protein isolate and soluble soybean polysaccharides. Structure, interfacial behavior and application on emulsions subjected to freeze-thawing. Food Hydrocoll..

[12] Ho K.K., Schroën K., San Martín-González M.F., Berton-Carabin C.C. (2017). Physicochemical stability of lycopene-loaded emulsions stabilized by plant or dairy proteins. Food Struct..

[13] Sun C., Liang B., Sheng H., Wang R., Zhao J., Zhang Z., Zhang M. (2018). Influence of initial protein structures and xanthan gum on the oxidative stability of O/W emulsions stabilized by whey protein. Int. J. Biol. Macromol..

[14] Fan L., Liu Y., Huang S., Li J. (2022). Effects of proteins on emulsion stability: The role of proteins at the oil–water interface. Food Chem..

[15] Guzey D., McClements D.J. (2007). Impact of electrostatic interactions on formation and stability of emulsions containing oil droplets coated by ß-lactoglobulin−pectin complexes. J. Agric. Food Chem..

[16] Perrechil F.A., Cunha R.L. (2013). Stabilization of multilayered emulsions by sodium caseinate and κ-carrageenan. Food Hydrocoll..

[17] Ray M., Rousseau D. (2013). Stabilization of oil-in-water emulsions using mixtures of denatured soy whey proteins and soluble soybean polysaccharides. Food Res. Int..

[18] Yan S., Xu J., Liu G., Du X., Hu M., Zhang S., Jiang L., Zhu H., Qi B., Li Y. (2022). Emulsions co-stabilized by soy protein nanoparticles and tea saponin: Physical stability, rheological properties, oxidative stability, and lipid digestion. Food Chem..

[19] Wei Z.H., Gao Y.X. (2016). Physicochemical properties of β-carotene bilayer emulsions coated by milk proteins and chitosan–EGCG conjugates. Food Hydrocoll..

[20] Zhao J., Xiang J., Wei T., Yuan F., Gao Y. (2014). Influence of environmental stresses on the physicochemical stability of orange oil bilayer emulsions coated by lactoferrin–soybean soluble polysaccharides and lactoferrin–beet pectin. Food Res. Int..

[21] Azarikia F., Abbasi S., Scanlon M., McClements D.J. (2017). Emulsion stability enhancement against environmental stresses using whey protein–tragacanthin complex: Comparison of layer-by-layer and mixing methods. Int. J. Food Prop..

[22] Chen L., Zhang L.W., Lei F.F., Zheng J.C., He D.P. (2022). Preparation of pumpkin seed oil O/W emulsion by whey protein isolate-xanthan gum compound emulsifier and its stability. China Oils Fats.

[23] Zang X., Wang J., Yu G., Cheng J. (2019). Addition of anionic polysaccharides to improve the stability of rice bran protein hydrolysate-stabilized emulsions. LWT.

[24] Donsì F., Wang Y., Huang Q. (2011). Freeze–thaw stability of lecithin and modified starch-based nanoemulsions. Food Hydrocoll..

[25] Hou P.P. (2020). Stability and Bioavailability of Whey Protein-Based Ginsenoside Rg3 Nanoemulsion.

[26] Liao Y., Sun Y., Peng X., Wang Q., Wu L., Yan S., Liu G., Zhu H., Qi B., Li Y. (2022). Preparation in vitro digestibility of perilla oil multilayer emulsion. Food Sci..

[27] Sun Y.X. (2020). Study on Construction, Stability and In Vitro Digestion of Camellia Oil Nanoemulsion.

[28] Sandra B., Stephan D. (2017). Saponins—Self-assembly and behavior at aqueous interfaces. Adv. Colloid Interface Sci..

[29] Zhang Z.P. (2021). Study on the Preparation of Egg White Protein-Chitosan Emulsion and the Stability of Loaded β-Carotene.

[30] Yi J., Li Y., Zhong F., Yokoyama W. (2014). The physicochemical stability and in vitro bioaccessibility of beta-carotene in oil-in-water sodium caseinate emulsions. Food Hydrocoll..

[31] Chang Y., McClements D.J. (2016). Influence of emulsifier type on the in vitro digestion of fish oil-in-water emulsions in the presence of an anionic marine polysaccharide (fucoidan): Caseinate, whey protein, lecithin, or Tween 80. Food Hydrocoll..

[32] Ozturk B., Argin S., Ozilgen M., McClements D.J. (2015). Nanoemulsion delivery systems for oil-soluble vitamins: Influence of carrier oil type on lipid digestion and vitamin D3 bioaccessibility. Food Chem..

[33] Liu W., Luo X., Wang J., Li Y., Feng F., Zhao M. (2021). Digestive behavior of unemulsified triglycerides with different chain lengths: In vitro dynamic and static simulated digestion models. LWT.

[34] Golding M., Wooster T.J. (2010). The influence of emulsion structure and stability on lipid digestion. Curr. Opin. Colloid Interface Sci..

[35] Zhang J., Bing L., Reineccius G.A. (2015). Formation, optical property and stability of orange oil nanoemulsions stabilized by Quallija saponins. LWT—Food Sci. Technol..

[36] Jiang L.Z., Qi Y., Ma C., Liu B., Wang Z., Li Y. (2018). Formation and stability of fish oil enriched biocompatible nano-emulsion. Trans. Chin. Soc. Agric. Mach..

[37] Pengon S., Chinatangkul N., Limmatvapirat C., Limmatvapirat S. (2018). The effect of surfactant on the physical properties of coconut oil nanoemulsions. Asian J. Pharm. Sci..

[38] Xu D., Yuan F., Wang X., Li X., Hou Z., Gao Y. (2010). The effect of whey protein isolate-dextran conjugates on the freeze-thaw stability of oil-in-water emulsions. J. Dispers. Sci. Technol..

[39] Zhang H., Fan Q., Li D., Chen X., Liang L. (2019). Impact of gum arabic on the partition and stability of resveratrol in sunflower oil emulsions stabilized by whey protein isolate. Colloids Surf. B Biointerfaces.

[40] Salvia-Trujillo L., Verkempinck S., Rijal S.K., Van Loey A., Grauwet T., Hendrickx M. (2019). Lipid nanoparticles with fats or oils containing β-carotene: Storage stability and in vitro digestibility kinetics. Food Chem..

[41] Xu X.F., Sun Q.J., McClements D.J. (2019). Enhancing the formation and stability of emulsions using mixed natural emulsifiers: Hydrolyzed rice glutelin and quillaja saponin. Food Hydrocoll..

[42] Brodkorb A., Egger L., Alminger M., Alvito P., Assunção R., Ballance S., Bohn T., Bourlieu-Lacanal C., Boutrou R., Carrière F. (2019). INFOGEST static in vitro simulation of gastrointestinal food digestion. Nat. Protoc..

